# A Novel Framework for* Ab Initio* Coarse Protein Structure Prediction

**DOI:** 10.1155/2018/7607384

**Published:** 2018-06-20

**Authors:** Sandhya Parasnath Dubey, S. Balaji, N. Gopalakrishna Kini, M. Sathish Kumar

**Affiliations:** ^1^Department of Computer Science & Eng., Manipal Institute of Technology, Manipal Academy of Higher Education, Manipal, Karnataka 576104, India; ^2^Department of Biotechnology, Manipal Institute of Technology, Manipal Academy of Higher Education, Manipal, Karnataka 576104, India; ^3^Department of ECE, Manipal Institute of Technology, Manipal Academy of Higher Education, Manipal, Karnataka 576104, India

## Abstract

Hydrophobic-Polar model is a simplified representation of Protein Structure Prediction (PSP) problem. However, even with the HP model, the PSP problem remains NP-complete. This work proposes a systematic and problem specific design for operators of the evolutionary program which hybrids with local search hill climbing, to efficiently explore the search space of PSP and thereby obtain an optimum conformation. The proposed algorithm achieves this by incorporating the following novel features: (i) new initialization method which generates only valid individuals with (rather than random) better fitness values; (ii) use of probability-based selection operators that limit the local convergence; (iii) use of secondary structure based mutation operator that makes the structure more closely to the laboratory determined structure; and (iv) incorporating all the above-mentioned features developed a complete two-tier framework. The developed framework builds the protein conformation on the square and triangular lattice. The test has been performed using benchmark sequences, and a comparative evaluation is done with various state-of-the-art algorithms. Moreover, in addition to hypothetical test sequences, we have tested protein sequences deposited in protein database repository. It has been observed that the proposed framework has shown superior performance regarding accuracy (fitness value) and speed (number of generations needed to attain the final conformation). The concepts used to enhance the performance are generic and can be used with any other population-based search algorithm such as genetic algorithm, ant colony optimization, and immune algorithm.

## 1. Introduction

The Protein Structure Prediction (PSP) problem is one of the major problems in the field of computational biology. The prediction of the native conformation of protein structure from its amino acid sequence is called PSP problem [1–4]. The solution to the PSP problem helps in understanding the molecular foundation of proteins in regulating life [5]. In wet lab, Nuclear Magnetic Resonance (NMR), X-ray crystallography (XC), and Cryoelectron microscopy are the methods used to solve the PSP problem [6]. Due to complexities and limitations of experimental methods, there is a huge gap in the number of reported protein sequences and their structures, and yet only 1% of structures are known for the reported protein sequences [7]. This calls for a computational solution to the PSP problem.

Computationally, the PSP problem is addressed by comparative modelling, threading, and* ab initio* approaches [8]. The first two approaches depend on known reference structure (template) to solve the PSP problem. Thus, the successes of these methods are limited by the template availability. The third approach predicts the native structure of the protein from its sequence; it is termed as* ab initio*. This method is devoid of a template and is better than the template-based methods [9]. One of the earliest* ab initio* approaches uses molecular mechanics to model protein structure. This method is based on simulating forces involved at the atomic level of amino acids such as covalent bonds, ionic bonds, hydrogen bonding, van der Waals interactions, and hydrophobic interactions. These molecular mechanics are applied in CHARMM, AMBER, and ECEPP [10, 11] energy functions and are found superior in modelling the fine conformation of proteins. But these energy functions are expensive in terms of both complexity and computational resources, making it infeasible even for the smallest protein sequence [12]. Besides, there are methods available based on interactions between specific pairs of amino acids with lower complexities such as Miyazawa-Jernigan (MJ) model [13–15] and Berrera energy model (BM model). Both these models use 20 x 20 energy matrix, and MJ uses effective contacts between all amino acid pairs, whereas BM uses van der Waals radii of alpha carbon and side-chain heavy atoms. Although these methods have reduced the computational complexity, a great amount of computing time is still required, which makes it computationally intractable for such elaborated energy function [16].

In 1985, Ken Dill [17] proposed a new computational perspective to lessen the modelling complexity and accelerate structure prediction to fill the sequence-structure gap, namely, the Hydrophobic-Polar (HP) model. Contrary to molecular mechanics and other modelling methods, the HP model considers hydrophobic interaction as the primary force involved in the folding process and yet was able to depict the natural folding patterns [18]. This is the most widely accepted approach to solve the PSP problem [19–21]. Although the HP model has reduced the computation time and complexity, it still falls under the category of the* NP-complete* problem [22]. In order to address* NP-hardness*, numerous researchers have worked with various population-based search algorithms such as Monte Carlo [23–25], genetic algorithm [19, 26–30], evolutionary programming [31], ant colony optimization [32–34], immune algorithm [35], constraint based chain growth algorithm [36], and hybrid of local search and evolutionary algorithms commonly called memetic algorithm [37–41]. The details on these algorithms and their performance are available in various review papers [18, 42].

Population diversity and selective pressure are two important parameters which control the performance of the aforementioned algorithms. But it has been observed that more often only one parameter is considered to reduce computing time of the PSP problem. As the PSP problem presents a rugged search space with more opportunity to get trapped in the local solution, undertaking of population diversity and selective pressure may result in better conformation with less computation (in few generations). Hence, this work integrates both these factors (i.e., population diversity and selective pressure) through a hybrid approach (evolutionary programming coupled with hill-climbing local search algorithm) for addressing PSP problem.

## 2. Material and Method

### 2.1. Hydrophobic-Polar Model

In the HP model, amino acids are classified into two major groups, namely, hydrophobic (H) and polar (P) [17]. The protein folding happens in an aqueous environment (cytoplasm), and the hydrophobic amino acids of the protein repel water, and this creates the driving force for folding. The hydrophobic amino acids arrange themselves to form a central hydrophobic core, and the polar amino acids are left on the surface to interact with water molecules. This can be explained by the oil-water behaviour [3], and the HP model mimics this.

The HP approach decomposed the PSP problem into three subproblems; first, defining a model to represent the protein structure referred to as conformation, second, defining the energy quantification based on amino acid properties that evaluate the modelled conformation, and, third, developing a search algorithm that can efficiently optimize conformation from a huge modelled space. In 1989 Lau and Dill proposed a lattice statistical mechanics model [3] used to represent the protein conformation commonly known as the lattice model or low-resolution model. In the lattice model, each amino acid is represented as a node of the lattice and two consecutive amino acids are connected through the lattice edge.

The modelled conformations are quantified using hydrophobic interaction (H-H contact) present in the lattice diagram. The hydrophobic interaction is defined by the topological distribution of hydrophobic amino acid; two H amino acids contribute to one unit of free energy value if H residues are adjacent (at the least distance) on lattice but nonconsecutive in a protein sequence. The free energy value is negative of H-H contacts. Concerning H-H contact, PSP is defined as maximization problem whereas with the free energy it is minimization problem. Formally, the PSP over HP model is defined as a triple (*S*, *f*, Ω), where *S* is a given search space (collection of different possible conformations), *f* is the objective function (in terms of hydrophobic contact or free energy value), which should be maximized or minimized, and Ω is the set of constraints that have to be fulfilled to obtain feasible solutions. The goal is to find a globally optimal solution, which is the solution *S*_*x*_ with the largest or smallest objective value under the condition that all constraints are fulfilled. Triple (*S*, *f*, Ω) is defined as follows:

*S*: it is a set of lattice conformations for given HP sequences.
*f*: it is an objective function to be maximized for hydrophobic contact or minimize the free energy value; here PSP is defined in terms of free energy value as
  (1) Minimize f=∑i,j:i+1<jcij·eij
where
  (2) cij=1,if  amino  acid  at  i  and  j  form  H−H  contact0,otherwiseeij=−1if  si=sj=H0,otherwise.

*Ω*: it is a set of the following constraints that need to be satisfied when modelling the PSP on a lattice:

(i) Self-avoiding walk (SAW): each amino acid must occupy only one lattice point, which no other amino acid can share.

(ii) Adjacent amino acids of the primary sequence must be at the unit distance.

### 2.2. Protein Structure Encoding

**Figure 1:**
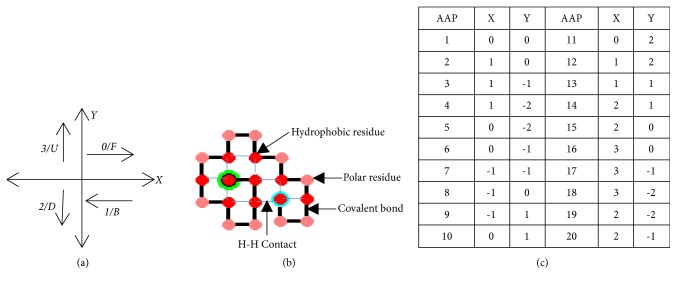
(a) Movement direction on XY axis for square lattice and (b) example conformation for 3H2P2(HP)H2P2(HP)H2PH, where encoding sequence is FDDBUBUUFUFDFDFDDBU and corresponding genotype (chromosome) is given in (c) amino acid position which is represented as “AAP” in the given sequence.

In this work, protein conformations are modelled using the nonisomorphic encoding proposed by Hoque et al. [43]. This encoding avoids the generation of isomorphic conformation; hence, the search space is free from degeneracy problem, where it consists of two different encodings corresponding to similar conformation. In such cases, if one conformation is kept at the center of the axis and the other rotating around it, in one quadrangle both structures will overlap. The existence of such conformation increases the use of the computational resources as they correspond to the similar structure and also makes the search space stagnate. A conformation C of n residues can be expressed in the form of movement direction such as C ∈ {F, B, U, D}^(n-1)^ and C ∈ {F, B, U, D, FU, BU, BD, FD}^(n-1)^ for two-dimension square and triangular lattice, respectively. F, B, U, D, FU, BU, BD, and FD are the movement direction to be followed on the Cartesian coordinate corresponding to* forward*,* backward*,* up*,* down*,* forward-up*,* backward-up*,* backward-down*, and* forward-down* direction. In the Cartesian coordinate these directional movements represent the single step move of (1, 0), (-1, 0), (0, 1), (0, -1), (1, 1), (-1, 1), (-1, -1), and (1, -1) respectively (Figure 1).

Also, nonisomorphic encoding ensures the 1:1 mapping for conformation and the encoded sequence. Hence, it performs the bijective mapping between genotype and phenotype.

## 3. Proposed Algorithm

**Figure 2:**
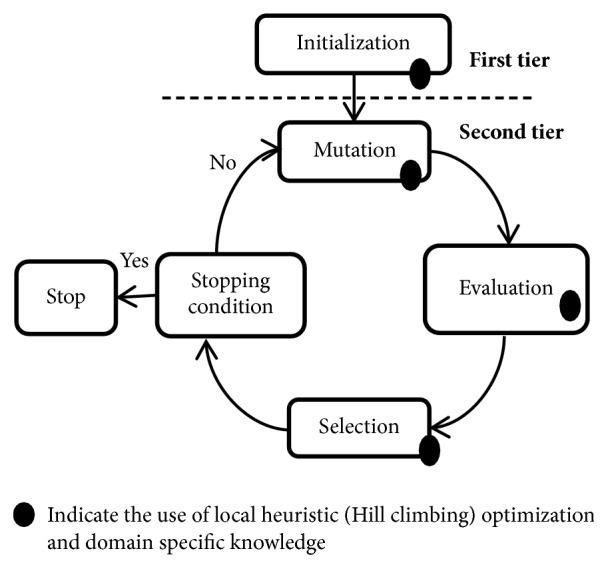
Layout of the proposed framework MA(EP+HC).

The proposed framework has a two-tier architecture, where first layer deals with the generation of the initial conformation and second layer improves the initial conformation by applying evolutionary operator mutation and selection. Besides exploitation and exploration of search space, we have considered the hybrid of local search with the evolutionary programming (MA(EP+HC)). The layout of the proposed framework is presented in Figure 2.

**Figure 3:**
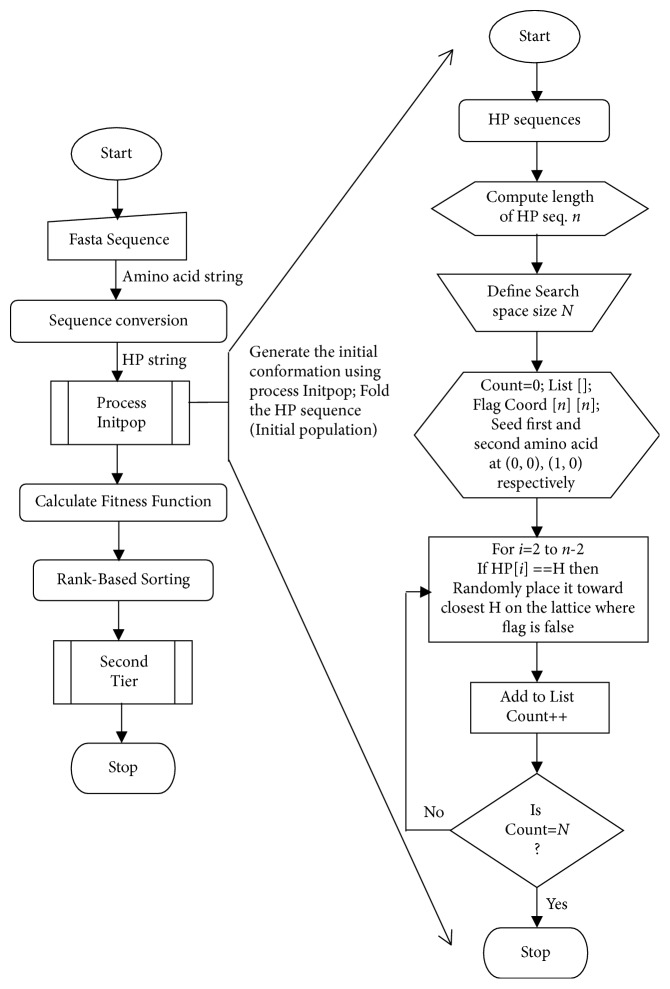
Expedition of first tier.

*First Tier*. The first tier deals with the sequence conversion and initial population generation. As shown in Figure 3, protein sequence is submitted in its “Fasta format” [44]. The input is converted into corresponding HP string by assigning the following subgroups H ∈ {A, G, I, L, M, F, P, W, V} and P ∈ {R, N, D, C, E, Q, H, K, S, T, Y} [45]. Further, HP sequence is used as input to generate the initial conformations. The initial conformations were obtained using the proposed initialization method developed by the hill-climbing algorithm to attain the valid conformation with minimal time and computation.

### 3.1. Hill Climbing

The hill-climbing (HC) algorithm is an iterative process to attain a solution to the NP-complete problem. It starts with one possible solution and iteratively improves it by satisfying the constraint of the problem. For instance, if 70% of current population converge to the same free energy value before reaching the termination condition, in such a case, HC employs diversity by random initialization whereas if the population space is too diverse, then the HC employs local search process to improvise the solution.

In this work, HC controls the trade-off between convergence and divergence of the population to avoid the trap of protein structure in the local optima. The pseudo code for used HC is given in Algorithm 1.

### 3.2. Evolutionary Programming

The evolutionary programming adopted in this research work is based on the first evolutionary program (EP) developed by L.J. Fogel (1962) [46]. “EP is derived from the simulation of adaptive behaviour in evolution.” This differs from other evolutionary algorithms such as GA, GP, and ES by following evolutionary behavioural model. The workflow of EP followed in the proposed work is presented in Algorithm 2. EP utilizes four main components of EAs: initialization, mutation, evaluation, and selection. Each of these components with reference to the PSP problem is discussed as follows.

#### 3.2.1. Population Initialization

The population initialization is the first step in all types of evolutionary algorithms as well as for stochastic search methods. For the PSP problem, protein conformation is individual, and a collection of conformations forms the population. The initial population generation is a random process, where initial conformations are generated; then they are subjected to confirm whether the generated conformation is valid or not.

For HP lattice-based PSP, conformation is valid if it follows the noncyclic move (self-avoiding walk). If not, it is discarded, and a new conformation is regenerated. This process inherently makes the generation of individuals computationally expensive. To overcome this limitation, which significantly slows down the search algorithm, we proposed a new initialization approach with the use of pointers, so that it ensures the generation of only SAW conformation and also provides the process to be significantly faster than random initialization.

Proposed initialization method is grounded on the fact that while folding, protein follows some specific folding pathways called folding rule. These folding rules are defined as follows: (a) Each amino acid searches for compatible neighbours and (b) each amino acid maintains its memory from previous interactions to preferred neighbour, thus resulting in a hydrophobic core. In the proposed method, sequence movement directions were obtained using random number generator function* rand()* which generates the number between 0 to 3 for square lattice four directional moves such that 0, 1, 2, and 3 refer to* forward* (F),* up* (U),* backward* (B), and* down* (D) move. Similar is the case with the triangular lattice, where* forward-up* (FU),* backward-up* (BU),* forward-down* (FD), and* backward-down* (BD) were selected by random numbers 6, 7, 8, and 9, respectively.

However, the residue movements were chosen based on the random number, but movement selection process is ruled by the hill-climbing (HC) algorithm, in contrast to random initializing. It is implemented using an array of pointer to list the availability of the lattice position.

The pointer holds the position of the lattice and, based on the availability of position, it is assigned its value; such that if the position is filled, then it updates the value as 1, whereas the available position has the value of 0. Further, the proposed method uses fixed position for the first two amino acids with the first at the center of the lattice at position of (0, 0) and second at the first forward move at (1, 0); then it follows* for* loop to generate the* l*-2 random number for the remaining residues. Concerning the random number and position move, associated coordinate value was assigned and stored in the array of a pointer which was used to intimate that the selected moves were SAW move or not. If not then it specifies the position x where the cycle appears. Further, it generates the* l*-2-*x* random number for the remaining residues and completes the conformation. A detailed comparison of the proposed method with the existing random initialization is presented in the Results and Discussion section. The entire process is represented as a flowchart (Figure 3).

*Second Tier*. The second tier of the proposed framework deals with the refinement of the initial conformation. Improvement of initial conformation has been attained with the hybrid of evolutionary programming (EP) and hill-climbing local search algorithm. EP works through initialization, mutation, evaluation, and selection operators of evolutionary algorithms [42, 47]. The success of EP is controlled by two main evolutionary operators: mutation and selection.

#### 3.2.2. Mutation Operators

**Figure 4:**
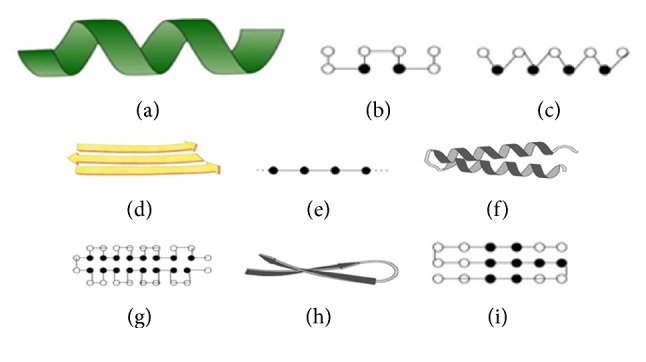
Protein secondary structures mapping with modelled structure, (a) helix, and (b)-(c) equivalent helix pattern on square and triangular lattice, respectively. (d) Sheet, (e) equivalent sheet for modelled conformation, (f) alpha-alpha motif, (g) equivalent motif for modelled conformation, (h) beta-beta motif, and (i) equivalent motif for modelled conformation.

**Figure 5:**
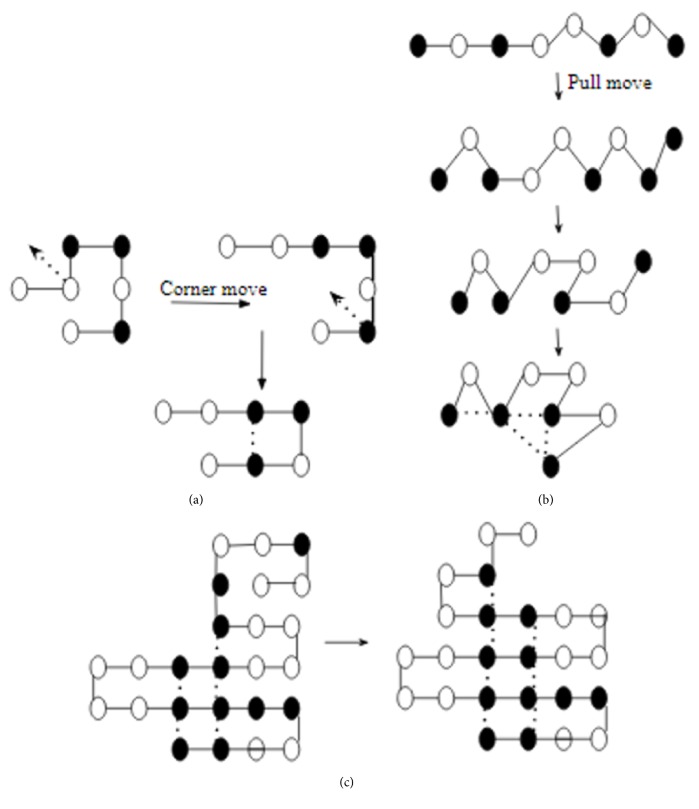
Set of mutation operators, namely, (a) corner, (b) pull, and (c) sample mutated conformation before with 5 H-H count (left) and after mutation 7 H-H count (right).

For EP, mutation is the only means to introduce variation in the population. Mathematically, mutation is defined as

(3) $$\begin{array}{c} x_{i}^{\prime }\left(\;t\;\right)=x_{i}\left(\;t\;\right)+\Delta x_{i}\left(\;t\;\right) \end{array}$$

where *x*_*i*_′(*t*) is the offspring created from parent *x*_*i*_(*t*) by introducing variation Δ*x*_*i*_(*t*) to *x*_*i*_(*t*). For the PSP problem, variation Δ*x*_*i*_(*t*) is introduced using one of the three types of mutation operators; helix, sheet, and corner/pull move over (square/triangular lattice), with the objective of increasing the H-H contact and secondary structural motif. The first two mutation operators are inspired by the protein secondary structure helix and sheets (Figure 4). The corner and pull move mutation (Figure 5) was adopted from the work of Hoque et al. [43]. The sequence patterns rule the selection of these mutation operators. For example, if the substring is P2HP2HP2, then it follows the downward directed helix (Figure 4(a)). It is observed that this results in the formation of the most compact mutation.

Similarly, the pattern HP2H2P2H follows the upward directed helix (Figure 4(b)). Contrary to this, if sequences have three or more repetitive residues, that follows sheet conformation (Figure 4(c)).

#### 3.2.3. Selection Operator

The primary objective of selection operator is to select the best individual, make multiple copies, and discard the rest keeping the population size constant. The selection is based on the relative fitness of individuals. Based on the score assigned to each, the following selection methods have been used.

*Tournament Selection*. It is used to facilitate the exploitation feature of EP. It is performed on the 20% of the population which have equal fitness value. Here, selected population is grouped into a set of *n*_*ts*_ individuals where *n*_*ts*_ < *n*_*s*_ (*n*_*s*_ is the total number of individuals in the population, and *n*_*ts*_ is 20% of ns). Every individual of a set is subjected to similar mutation, and the best performing is returned for further optimization.

*Rank-Based Selection*. Here, the selection is based on the relative ranking. Hence, the selection is independent of actual fitness values, with the advantage that the best individual will not dominate in the selection process. Nondeterministic linear sampling selects an individual, *x*_*i*_, such that *i* ~ ∪(0, ∪(0, *n*_*s*_ − 1)), where the conformations are sorted in decreasing order of their fitness values. It ensured unbiased selection and used over 75% of the population.

It is also assumed that the rank of the best individual is 0 and that of the worst individual is *n*_*s*_ − 1. The linear ranking assumes that the best individual creates *λ*, offspring, and the worst individual *λ*′, where 1 ≤ *λ* ≤ 2 and *λ*′ = 2 − *λ*. The selection probability of each individual is calculated as

(4) $$\begin{array}{c} \varphi_{s}\left(\;x_{i}\left(\;t\;\right)\;\right)=\frac{1-e^{-f_{r}\left(x_{i}\left(t\right)\right)}}{\beta } \end{array}$$

where *f*_*r*_(*x*_*i*_) is the rank of *x*_*i*_ (i.e., the conformation's position in the ordered sequence of individuals) and *β* is the normalization constant.

*Elitism*. Elitism refers to the process of ensuring that the best individuals of the current population survive to the next generation. This ensures retaining the best conformation. The probability of selection in this method is 5%.

*Aging Operator*. If particular conformation is elitist for consecutive twenty-five generations and if they are not enhancing the fitness values through evolutionary operators, then they are called aged individuals. Further tournament selection is performed among the aged individuals of equal fitness. Based on the resultant value of this operator, it will either be elitist or discarded. This helps in maintaining the diversity and exclusion from getting trapped in local minima.

#### 3.2.4. Other Algorithmic Features Considered

*Population Size*. The larger the population size, the more the diversity, thereby improving the exploration abilities of the population. However, the higher the population size, the more the computational complexity per generation. While the execution time per generation increases, it may be the case that fewer generations are needed to locate an acceptable solution. The considered population size in this work is 200 as followed in many of the state-of-the-art works [47].

*Fitness Function*. The fitness function is defined as follows:

(5) Maximize f=∑i,j:i+1<j;si=sj=Hcijcij=1,if  residue  at  i  and  j  are  at  adjacent  point  on  the  lattice0,otherwise.

It returns the number of H-H count present in the given conformation.

*Stopping Condition*. This is used to bind the number of generations allowed to execute for EA and execution time. Further, convergence criterion is used to detect if the population has converged. In the context of the PSP problem, convergence is defined as a state when there is no increase in the fitness value of conformation or the state when it ends in a similar conformation.

The test HP sequences (Table 1) were taken from the study of Garza-Fabre et al. [18]. Initially, conformations are built using random generation technique. Later, conformations are built using proposed approach for the same test beds. The implementations of both random initialization and proposed initialization were carried out on the same machine. The effect of the proposed approach is measured using H-H count value and the number of valid conformations generated. The noncyclic conformations were called valid conformation satisfying the SAW constraint of Dill's HP model. The evaluation would be equal to the level of the phenomenon (H-H count) after the treatment (proposed initialization approach) minus the level of the phenomenon (H-H count) before the treatment (random initialization).

The executable code is implemented using Visual C++ on Microsoft Visual Studio 2012 where conformations are visualized using OpenGL graphics. The complete program is available at www.abinitio-hp-psp.com.

## 4. Results and Discussion

To evaluate the efficiency of the proposed initialization approach, both proposed and random initialization programs have been executed to generate 100 conformations for the benchmark sequences [18] presented in Table 1. Both of the initialization programs were implemented locally on the same system. The results of initialization algorithms are shown in Table 2. The comparative evaluation for random versus proposed initialization approaches are as shown in Table 2.

### 4.1. Based on Methodology

In random initialization method, for each constituting residue of a given sequence, first, it generates random numbers between 0 and 3. Numbers 0, 1, 2, and 3 represent the forward (F), back (B), up (U), and down (D) movements on the 2D lattice, respectively, and are used to encode the conformation. In this approach, first, it assigns the move, i.e., position in the 2D space, and then the conformation is subjected to the SAW evaluation.

Meanwhile, in the proposed approach, it assigns the moves using 2D flag array. Size of array is the square value of sequence length. Initially, all values of the array are set to false, and once the coordinate is assigned, it turns to true. Furthermore, for each assignment of movement, it checks the array, and if it is occupied (array value is true), then it cancels the movement and attempts with other positions. This has resulted in the reduction of time to generate the conformations, as, instead of cancelling the complete assignment, it checks the alternate position to fill out the conformation. It has been observed that, with random initialization, most of the conformation fails to satisfy the SAW constraint. The values for a number of SAW(*N*_*v*_) and non-SAW(*N*_*i*_) conformations generated in one run are depicted in Table 2.

**Figure 6:**
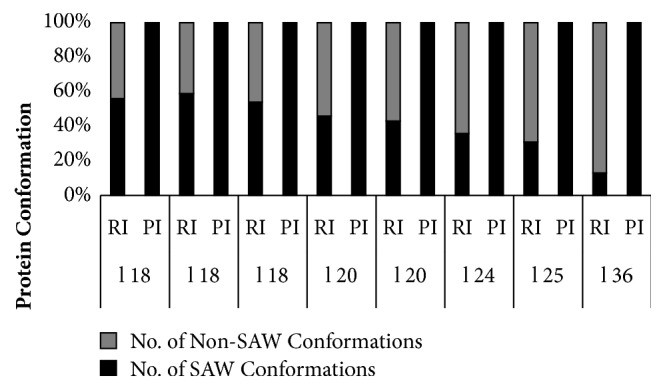
Graph for the number of SAW (noncyclic) and Non-SAW (cyclic) conformations generated using random (RI) and proposed initialization (PI) method. “l” refers to the length of the sequence.

It has been observed that, with random initialization, the number of SAW conformations is inversely proportional to the length of the given sequences (Figure 6), whereas the proposed initialization approach generates only valid conformations(*N*_*k*_).

The proposed initialization approach has completely removed the generation of invalid conformations, whereas random approach fails to generate all valid conformations. Moreover, as the sequence length increases, the number of invalid conformations increases and valid structures decrease with random initialization. This results in multiple executions of the initialization function to attain the defined number of initial conformations, which serve as input for further application of evolutionary operators, mutation, and crossover. Contrary to this, the proposed initialization method has generated only SAW conformations, resulting in a reduction in uses of computational resources as well as time.

### 4.2. Based on Free Energy

Free energy (E*∗*) value is a quantitative parameter that evaluates the predicted structure for protein. According to thermodynamic law, a particle in its native state has the lowest free energy. As stated in Section one, for the PSP problem, the higher the H-H count, the lower the energy value and, hence, the more optimal the structure.  Table 3 represents the lowest free energy value obtained with a random and proposed algorithm implemented with 100 generations having 100 as the population size (100 x100 conformations).

**Figure 7:**
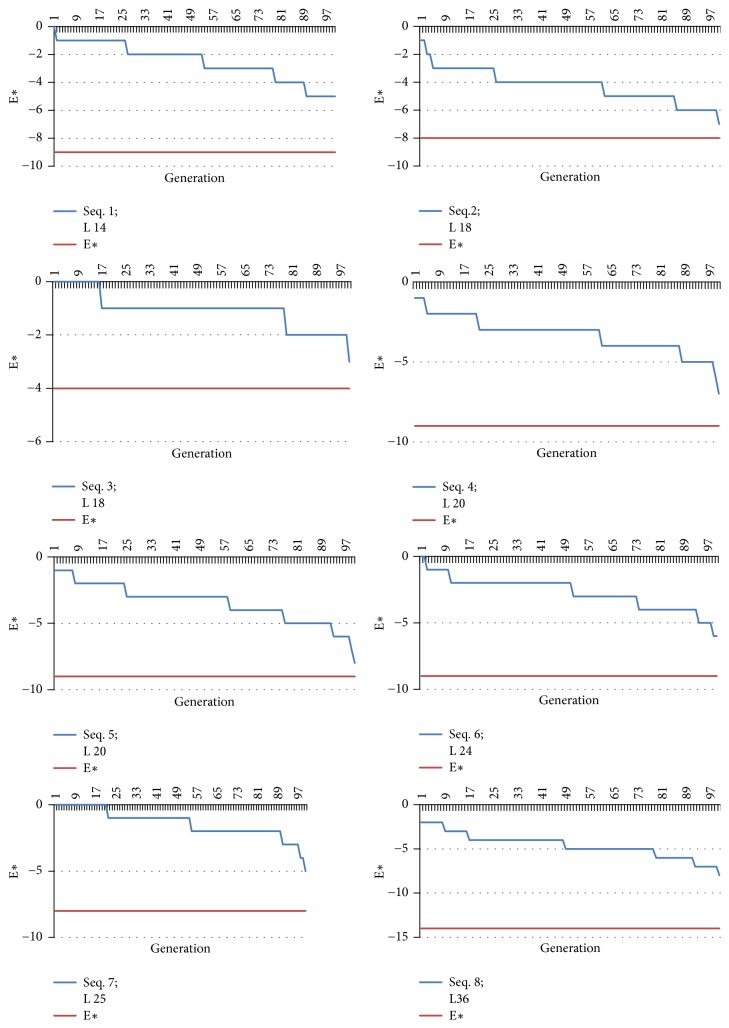
Line graph for optimum free energy values E*∗* and energy value obtained with the proposed initialization approach with 100 conformations in each generation for the benchmark sequences used in this study.

In Table 3, E*∗* is the lowest free energy value reported in the state-of-the-art research work of Islam and Chetty [47].  Figure 7 shows the free energy variation for each tested sequence over the 100 generations through proposed approach. This study has been done to find out if there is any trend in the value of free energy to reach the optimum E*∗*. It has been observed that as the generation count increases, the gap between E*∗* and the obtained E reduces, which results in a significant drop in the required number of generations needed to attain E*∗*.

**Figure 8:**
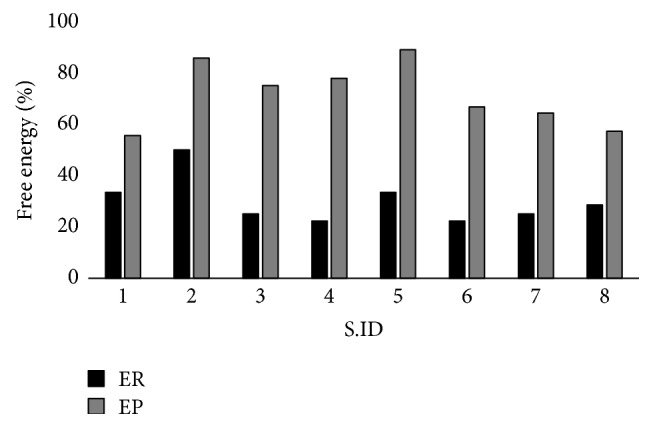
Line graph to represent the closeness of free energy value obtained with the random E^R^ and proposed E^P^ initialization techniques with respect to reported free energy E*∗* value [18].

Moreover, proposed approaches have nearly reached the reported optimum values as shown in Figure 7. In this study, the value of E*∗* has been considered as 100% and then evaluated with the results of proposed and random initialization approach to evaluate how far or near it is with the E*∗* in percentage. This result is presented in Figure 8.

The EP outperforms ER in all the tested sequences concerning free energy value. EP values are closer to the E*∗* as shown in Figure 8. On the other hand, the free energy values obtained with the ER fails to reach 50% of the optimum value. Hence, conformations generated using random generation require more use of evolutionary operators to attain the optimum conformation whereas conformation generated using proposed initialization needs fewer operators. This contributes to an immense drop in time to reach the optimum conformation.

### 4.3. Number of Hydrophobic Residues and Free Energy Values

This analysis has been done to find if there is any trend in folding and free energy values depending on the number of hydrophobic (H) residues. It has been observed that the higher the hydrophobic residue percentage, the lower the free energy value as shown in Table 3. Sequences 1, 2, and 3 have the same residue count of 18, but the percentage of H residue is higher in 1 and 2 between 57 and 61% whereas in 3 it is 33%. This may result in the difference in the value of free energy. The similar pattern has been found for sequences 3, 4, and 5 where each of these sequences has the same length and nearly the same hydrophobic residue percentages, which has resulted in identical free energy values of -9. This concept can be used to predict the number of optimum hydrophobic contacts for the novel protein sequences, and it can also be helpful in determining the protein core formation and distribution of residues in the 3D structure.

### 4.4. Based on the Convergence Rate

The convergence rate is another parameter to evaluate the performance of the proposed approach. It is given in terms of percentage and calculated using formula given in

(6) Convergence  rate%=1−Fitness  Ex−E∗E∗×100.

The above formula is adopted from the work of Paul et al. [50]. They proposed the novel population initialization technique for the traveling salesman problem using a genetic algorithm and performed the characteristic performance evaluation using (6). With regard to convergence, the proposed approach outperforms the random initialization method as shown in Table 3, where convergence rate for the proposed algorithm in average is 71% whereas random approach average convergence rate is 30% as the higher the convergence rate is, the faster it is to reach the optimum conformation. The maximum and minimum values for proposed convergence are 88.9% for sequence 5 and 55.6% for sequence 1 and in case of random initialization highest convergence value is 50%, and lowest is 22.3% as shown in Table 3.

Thus, at least 55.6% of convergences have been obtained with the initial population for solving the PSP problem, with proposed initialization in the initial phase which contributes to the reduction in the consumption of computational resources and computation time by early convergence towards the optimum solution. Further, the average convergence of proposed approach for other instances lies between 88.9% and 55.6% which indicates that the initial set of solutions have an excellent collection of varying conformation. This could help in the exploration of wide search space and avoid the possibility of getting trapped in local solution.

### 4.5. Square Lattice Model

The proposed framework has been evaluated with five state-of-the-art approaches, namely, MMA [37], ACO [34], IA [35], GGA [18], and CMA [47]. The proposed framework is implemented with an initial population of 100 conformations. These conformations are generated using the first layer of the framework, where it employs domain-specific knowledge to compute the H-core in the initial conformation. Later, these conformations are passed to the second tier where various mutation operations are applied to improvise the fitness function values. The proposed framework is run to achieve the reported optimum fitness values, with a minimum of 50 runs and maximum of 500.

**Figure 9:**
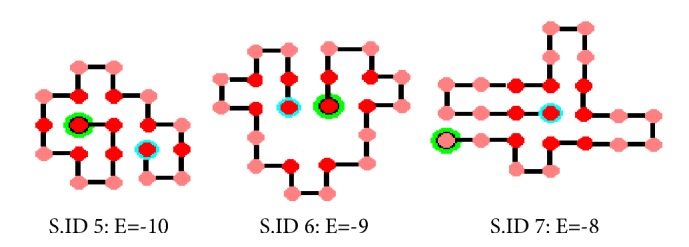
Optimal conformation obtained with MA(EP+HC) for sequences no. 5, 6, and 7.

Every run is independent of each other and executed on the same system. Results obtained with the proposed framework and other EAs have been presented in Table 4.  Figure 9 represents the conformation for sequences 5, 6, and 7.

Although most of the EAs show similar fitness values as compared with the proposed framework values, the difference lies in the population size and the number of generations used. CMA [47] uses a population size of 100, whereas, in EDA [47], the population size is 5000, which is 50 times larger than the CMA population size. The result shows that the MA(EP+HC) performs consistently and achieves equivalent fitness values in fewer generations and hence reduces the computation time. Note that Table 4 includes a column for generation count of MA(EP+HC) algorithm, which is needed to attain the optimum value. As this value is not available in any related work, hence no comparison could be made based on the generation count.

### 4.6. Triangular Lattice Model

For triangular lattice, this study has been carried out using benchmark sequences taken from the work of Islam and Chetty [47]. In this implementation, we have compared the performance of MA(EP+HC) with HGA [48], ERS-GA [39], HHGA [39], TS [49], CMA [47], and OSSGA [40] and results are summarized in Table 5. It has been observed that the MA(EP+HC) has obtained the lowest free energy (Table 5) for the test sequence in experiment compared to the previous state-of-the-art approaches.

To check the efficiency of MA(EP+HC) for longer sequences, we have extended our study for test sequences B9 to B11 (Table 5), and these test sequences are taken from the work of Islam and Chetty [47]. However, none of the other works has considered sequences longer than 64 for triangular lattice. In our execution, MA(EP+HC) ran with a population size of 100 similar to CMA. As each of the compared works executed on different systems with different configurations, comparing the results based on time is not a correct approach. To overcome such problems in the future, we are providing the complete developed program as supplementary material with this paper.

So, in future, if anyone wants to do a comparative study they can execute the program on their system and evaluate the results based on the execution time. This could result in a precise parameter to assess further work in future. Also, it contributes to other research areas including use of EAs as in drug designing, protein engineering, the study of the n-body problem, etc.

### 4.7. Additional Biological Sequences

To verify the consistency of the proposed MA(EP+HC) framework, this study has also been extended for the experimentally derived proteins. To carry out this study, we retrieved the proteins from the Protein Data Bank (PDB) [44].

**Figure 10:**
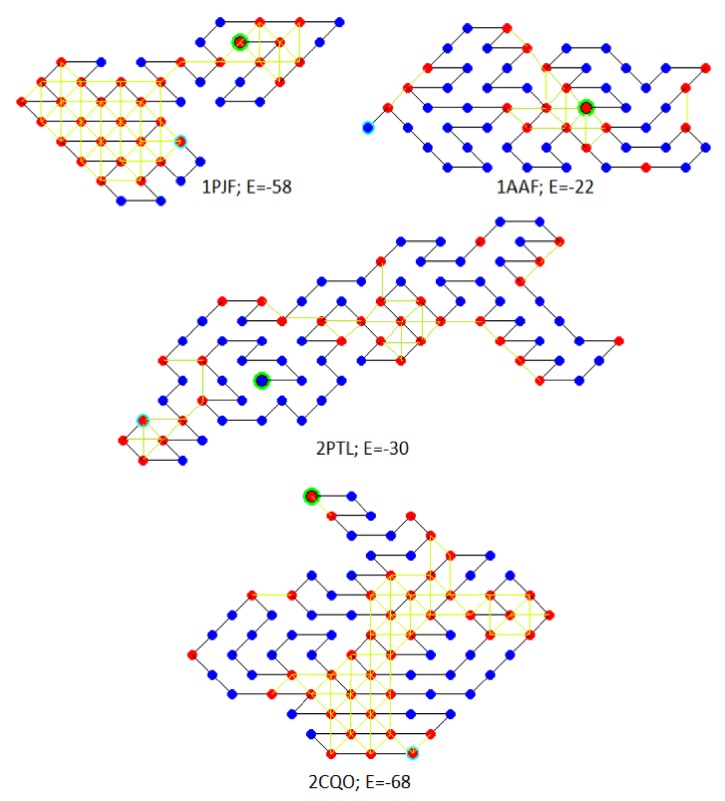
The figure depicts the model conformation for sequence listed in Table 6. The hydrophobic/polar residue is represented using red/blue filled circle, and the first residue is encircled with green and ends with sky blue. An adjacent connection is represented using black line and H-H contact with the yellow line. The free energy is equal to the number of yellow lines.

To perform the prediction, first obtained sequences were converted into their HP sequence (Table 6). Later they folded using the MA(EP+HC) framework. The optimal conformations obtained with proposed MA(EP+HC) framework are shown in Figure 10. The structures obtained with MA(EP+HC) framework have exhibited favourable performance. Also, the proposed algorithm has outperformed the result of GAT [29].

## 5. Conclusions and Future Work

The proposed initialization approach has produced near-optimal free energy conformation for the tested benchmark sequences when compared with the random initialization in the initial stage of an evolutionary program. This has also reflected in the convergence rate (71%) of the proposed approach that is 40% faster than the random initialization approach.

Also, the proposed framework was evaluated for experimentally derived protein sequence and has been found to be more efficient for triangular lattice. In the square lattice, the proposed framework has performed similarly to other search algorithms. This study has been limited to the sequence length of 100. But it can be extended for longer protein sequences (sequence length more than 100), and we are working in this direction. Overall, the concept of domain-specific intelligent initialization and mutation can be used in other domain-specific NP-problems.

## Figures and Tables

**Algorithm 1:**
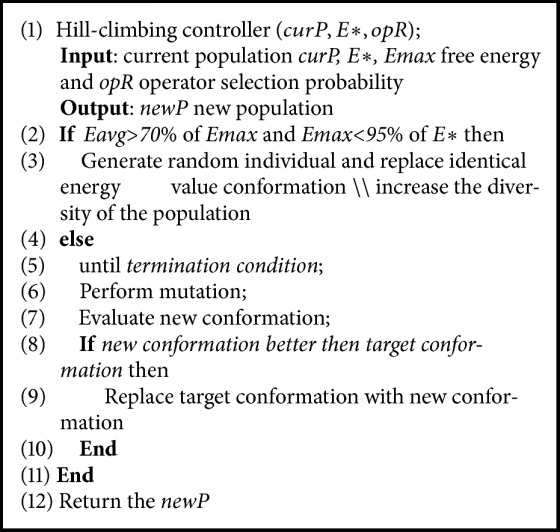
The pseudocode for hill-climbing controller.

**Algorithm 2:**
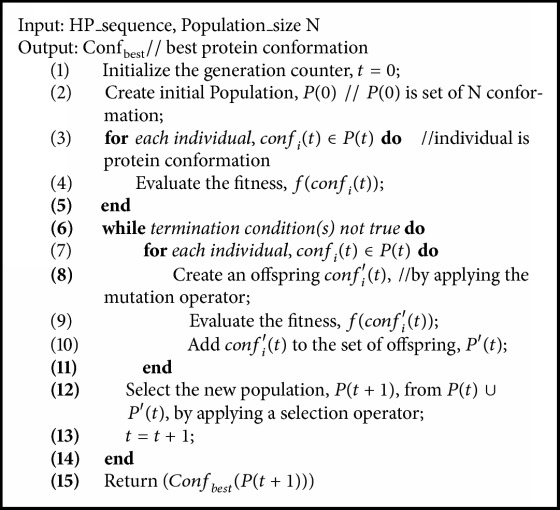
The pseudocode for evolutionary programming.

**Table 1:** Benchmark sequence to test on 2D square lattice [18].

**Seq. No.**	**Sequences**	**Seq. length**	**Max H-H**
1	PH2PHP3HP2HP5H	18	-9
2	HPHP3H3P4H2P2H	18	-8
3	2H5P2H3PH3PHP	18	-4
4	HPH2P2HPH2PHP2H2PHPH	20	-9
5	3H2P2(HP)H2P2(HP)H2PH	20	-10
6	2H2P6(H2P)2H	24	-9
7	2PH2P3(2H4P)2H	25	-8
8	3P2H2P2H5P7H2P2H4P2H2PH2P	36	-14

**Table 2:** Results of initialization algorithm.

S.ID	L	Random initialization			Proposed initialization	
		*H*_*R*_	*N*_*v*_	*N*_*i*_	*H*_*k*_	*N*_*k*_
1	18	3	56	44	5	100
2	18	4	59	41	7	100
3	18	1	54	46	7	100
4	20	2	46	54	7	100
5	20	3	43	57	8	100
6	24	2	36	64	6	100
7	25	2	31	69	5	100
8	36	4	13	87	8	100

*H*_*R*_, *H*_*k*_: H-H count value obtained with random and proposed initialization respectively; *N*_*v*_: number of valid conformations; *N*_*i*_: number of invalid conformations; *N*_*k*_: number of conformations generated using proposed initialization.

**Table 3:** Free energy value comparison of first tier.

S. ID	E*∗*	E^R^	E^P^	E^%^ = (E/E*∗*)*∗*100		H^%^	C^R%^	C^P%^
				E^R%^	E^P%^			
1	-9	-3	-5	33.3	55.6	57	33.4	55.6
2	-8	-4	-7	44.4	87.5	61	50	85.7
3	-4	-1	-3	25	175	33	25	75
4	-9	-2	-7	22.2	77.8	50	22.3	77.8
5	-9	-3	-8	33.3	88.9	50	33.4	88.9
6	-9	-2	-6	22.2	66.7	42	22.3	66.7
7	-8	-2	-5	25	62.5	36	25	64.3
8	-14	-4	-8	28.6	57	44	28.6	57.2

E*∗*: lowest free energy value from [35]; E^R^, E^P^: lowest free energy value obtained with random and proposed approach, respectively; E%: % of E value with respect to optimum E*∗* value; H% = (n_h_/*L*)*∗*100 of H element in given sequence; n_h_: number of hydrophobic residues; C^R%^, C^P%^: convergences rate for H-H contact with random and Knowledge based approach, respectively.

**Table 4:** Optimum energy value E*∗* obtained with various search algorithms on 2D square lattice model.

S. ID	MMA [37]	ACO [34]	IA [35]	GGA [18]	CMA [47]	MA(EP+HC)	G_c_
1	-9	N/A	-9	-9	N/A	-9	98
2	-8	N/A	-8	-8	N/A	-8	361
3	-4	N/A	-4	-4	N/A	**-7**	54
4	-9	-9	-9	-9	N/A	-9	342
5	-10	N/A	-10	-10	N/A	-9	400
6	N/A	-9	-9	-9	-9	-9	249
7	-8	-8	-8	-8	-8	-8	301
8	-14	-14	-14	-14	-14	14	435

N/A: not used in the study; G_c_: number of generations needed to attain the optimum value.

**Table 5:** Benchmark HP sequences [47] and their respective free energy E*∗* value with the various methods on the 2D triangular lattice model.

S.ID	*L*	Sequence	HGA [48]	ERS-GA [39]	HHGA [39]	TS [49]	CMA [47]	OSSGA [40]	MA(EP+HC)
B1	20	(HP)^2^PH(HP)^2^(PH)^2^HP(PH)^2^	-15	-15	-15	-15	-15	-15	**-17**
B2	24	H^2^P^2^(HP^2^)^6^H^2^	-13	-14	-14	**-17**	**-17**	**-17**	-15
B3	25	P^2^HP^2^(H^2^P^4^)^3^H^2^	-10	-11	-11	-12	-12	--12	**-14**
B4	36	P(P^2^H^2^)^2^P^5^H^5^(H^2^P^2^)^2^P^2^H(HP^2^)^2^	-19	-22	-22	-24	-24	-24	**-28**
B5	48	P^2^H(P^2^H^2^)^2^P^5^H^10^P^6^(H^2^P^2^)^2^HP^2^H^5^	-32	-34	-34	-40	-40	-43	**-45**
B6	50	H^2^(PH)^3^PH^4^PH(P^3^H)^2^P^4^(HP^3^)^2^HPH^4^(PH)^3^PH^2^	-23	-32	-32	N/A	-41	-41	**-38**
B7	60	P(PH^3^)^2^H^5^P^3^H^10^PHP^3^H^12^P^4^H^6^PH^2^PHP	-46	-62	-62	-70	-70	-70	**-90**
B8	64	H^12^(PH)^2^((P^2^H^2^)^2^P^2^H)^3^(PH)^2^H^11^	-46	-51	-51	-50	-75	-74	**-76**
B9	85	H^4^P^4^H^12^P^6^H^12^P^3^H^12^P^3^H^12^P^3^H(P^2^H^2^)^2^(PH)^2^	N/A	N/A	N/A	N/A	N/A	N/A	**-115**
B10	100a	P^6^HPH^2^P^5^H^3^P^5^HPH^2^P^4^H^2^P^2^H^2^P^5^HPH^10^PH^2^PH^7^P^11^H^7^P^2^HPH^3^P^6^HPH^2^	N/A	N/A	N/A	N/A	N/A	N/A	**-70**
B11	100b	P^3^H^2^P^2^H^4^P^2^H^3^(PH^2^)^2^PH^4^P^8^H^6^P^2^H^6^P^9^HPH^2^P^11^H^2^P^3^HPH^2^PHP^2^HPH^3^P^6^H^3^	N/A	N/A	N/A	N/A	N/A	N/A	**-72**

Bold face values indicate the lowest free energy; N/A: not applicable in respective study.

**Table 6:** Tested biological sequence taken from PDB [44].

PDB ID	*L*	Sequence	GAT [29]	MA(EP+HC)
1PJF	46	GVIDTSAVESAITDGQGDMKAIGGYIVGALVILAVAGLIYSMLRKA HHHPPPHHPPHHPPHPHPHPHHHHPHHHHHHHHHHHHHHPPHHPPH	-25	-58
1AAF	55	MQRGNFRNQRKIIKCFNCGKEGHIAKNCRAPRKRGCWKCGKEGHQMKDCTERQAN HPPHPHPPPPPHHPPHPPHPPHPHHPPPPHHPPPHPHPPHPPHPPHPPPPPPPHP	-15	-22
2PTL	78	ENKEETPETPETDSEEEVTIKANLIFANGSTQTAEFKGTFEKATSEAYAYADTLKKDNGEYTVDVADKGYTLNIKFAG PPPPPPHPPHPPPPPPPHPHPHPHHHHPHPPPPHPHPHPHPPHPPPHPHPHPPHPPPPHPPPHPHHPPHPPHPHPHHH	-25	-30
2CQO	119	GSSGSSGMNSGRPETMENLPALYTIFQGEVAMVTDYGAFIKIPGCRKQGLVHRTHMSSCRVDKPSEIVDVGDKVWVKLI GREMKNDRIKVSLSMKVVNQGTGKDLDPNNVIIESGPSSG HPPHPPHHPPHPHPPHPPHHHHPPHHPHPHHHHPPPHHHHPHHHPPPPHHHPPPPHPPPPHPPHPPHHPHHPPHH HPHHHPPHPPPPHPHPHPHPHHPPHPHPPHPHPPHHHPPHHPPH	-45	-68

*Note*. Column sequence contains protein sequence and its HP sequence.

## Data Availability

The data used to support the findings of this study are available from the corresponding author upon request. The complete program is available at www.abinitio-hp-psp.com.
